# Consensus-Level and Cluster-Adjusted Evaluation of a Large Language Model for Diagnostic Extraction from Musculoskeletal Radiology Reports

**DOI:** 10.3390/diagnostics16111590

**Published:** 2026-05-22

**Authors:** Wolfram A. Bosbach, Elham Montazeri, Jan F. Senge, Claus Beisbart, Milena Mitrakovic, Suzanne E. Anderson, Eugen Divjak, Gordana Ivanac, Thomas Grieser, Marc-André Weber, Hatice Tuba Sanal, Keivan Daneshvar

**Affiliations:** 1 Department of Nuclear Medicine, Inselspital, Bern University Hospital, University of Bern, 3010 Bern, Switzerland; 2 Department of Mathematics and Computer Science, University of Bremen, 28359 Bremen, Germany; janfsenge@uni-bremen.de; 3 Dioscuri Centre for Topological Data Analysis, Institute of Mathematics, Polish Academy of Sciences, 00-656 Warsaw, Poland; 4 Institute of Philosophy, University of Bern, 3012 Bern, Switzerland; 5 Department of Diagnostic, Interventional and Paediatric Radiology (DIPR), Inselspital, Bern University Hospital, University of Bern, 3010 Bern, Switzerlandkeivan11@hotmail.com (K.D.); 6 School of Medicine, Sydney Campus, University of Notre Dame, Broadway, P.O. Box 944, Sydney 2007, Australia; 7 Department of Diagnostic and Interventional Radiology, University Hospital “Dubrava”, University of Zagreb School of Medicine, 10000 Zagreb, Croatia; 8 Department of Diagnostic and Interventional Radiology, University Hospital Augsburg, 86156 Augsburg, Germany; 9 Institute of Diagnostic and Interventional Radiology, Pediatric Radiology and Neuroradiology, University Medical Center Rostock, 18057 Rostock, Germany; marc-andre.weber@med.uni-rostock.de; 10 Radiology Department, Gülhane Training and Research Hospital, University of Health Sciences, 06010 Etlik, Ankara, Türkiye

**Keywords:** automation in radiology, LLM in medicine, workflow optimisation, MSK radiology

## Abstract

**Purpose:** Large language models (LLMs) may reduce administrative workload in radiology by automating structured diagnostic extraction from text reports. This study evaluates the accuracy of ChatGPT-4.0 when extracting correct diagnoses from musculoskeletal (MSK) radiology text reports, and compares its performance with that of experienced human readers, using cluster-adjusted and consensus-level analyses. **Materials and Methods:** Twenty-three multimodal MSK imaging cases (X-ray, ultrasound, CT, and MRI) were analysed. Ten human readers and ChatGPT-4.0 (10 independent iterations) provided primary (1st) and secondary (2nd) diagnoses from six predefined options. We analysed data at the individual-reader level using cluster-adjusted generalised estimating equations (GEE) and at the case level using majority consensus with exact McNemar testing. Within-case (α_case) and within-reader (α_reader) correlations and design effects were calculated to assess clustering and implications for sample size. **Results:** For 1st diagnoses, AI accuracy was 0.957 (95%–CI 0.922–0.976) versus 0.865 (95%–CI 0.815–0.903) for human readers (absolute difference −0.091; OR 3.43, 95%–CI 1.07–11.02; *p* = 0.038). Within-case correlation (α case = 0.247) exceeded within-reader correlation (α reader ≈ 0); this resulted in a design effect of 5.7 and an effective sample size of 80.7. At the consensus level, discordance occurred in 2/23 cases (8.7%), with no significant difference between methods (McNemar *p* = 1.00). When 1st and 2nd diagnoses were combined, both systems achieved 23/23 correct consensus classifications. Interrater reliability between AI and human classifications was almost perfect (Gwet’s AC1 = 0.836–0.927). **Conclusions and Key points:** In this structured MSK text-report setting, ChatGPT-4.0 achieved diagnostic accuracy comparable to that of experienced radiologists, with modest individual-reader advantages that disappeared under consensus aggregation. Clustering analysis indicates that variability is primarily case-driven, suggesting that future validation studies will benefit more from expanding case numbers than reader numbers. Our data suggest that large performance divergences between AI and human consensus are unlikely in similar structured diagnostic contexts.

## 1. Introduction

Today, healthcare staff spend a substantial portion of their working hours on administration and documentation (often >15% [1]). This is true for doctors, nurses, and non-medical staff, irrespective of medical speciality. Patients would directly benefit from reducing the time spent on paperwork, provided the quality of patient outcomes is maintained. At the same time, the predicted future increase in demand for radiological services might not be met without an increase in capacity [2,3]. Novel large language models (LLMs, e.g., [4]) promise to provide the necessary toolsets for this transformation of medical processes. In the ideal case, LLMs will increase patient throughput while simultaneously improving the quality of patient outcomes.

Artificial intelligence research has evolved substantially since its early conceptual foundations [5], with recent advances enabling practical applications in radiology. The width of the literature today in our field would be beyond the scope of this introductory section. The growth of data in size, connectivity between datasets, and speed of end-user devices has led to the point where radiology and other specialities are likely at a potential transition point [6]. Traditionally, humans assess the image output of the various radiological imaging modalities and type their findings/impressions by hand. However, now, commercial software solutions incorporating artificial intelligence (AI) components are being tested. One example is Aidoc Medical’s software for analysing computed tomography (CT) scans (Tel-Aviv, Israel [7]). Other systems are, e.g., the software by Gleamer (Paris, France [8,9]) and AZmed (France, Paris [10,11,12]), which are designed to detect abnormalities in chest X-rays [13] and bone fractures. Tests show that AI software now exhibits reasonable performance in clinical applications, not limited to laboratory conditions. In addition, the mentioned AI software is becoming increasingly price-competitive. Both factors have made an uptake into clinical processes increasingly likely. Whether human radiologists will soon work in hybrid human-AI settings remains to be seen.

The field of LLM application testing is expanding rapidly in radiology [14,15,16], and likewise also, e.g., in nuclear medicine [17]. A typical test for showcasing LLM performance is now the simulation of board exams [18]. The reproducible nature of board exams and the ability to repeat them in the future allow tracking the continuous improvement of LLM systems. Improvements of newer LLM versions have been documented in the past, e.g., for American College of Radiology (ACR) Diagnostic in Training Exam (DXIT) practice questions [19]. When applied appropriately, LLMs have demonstrated the ability to write radiology reports for chest X-rays [20,21,22]. This step from mere image analysis to report writing is of substantial importance in radiology as the main form of communication with referring physicians is still via the written text report. The difficulty in writing adequate reports can be seen, e.g., in the determination and expression of perceived probability in text [23]. If LLMs are supposed to find their way into clinical routine, they will have to master this and multiple other nuances of linguistic expressions. Clinical decision making in interventional radiology [24] and also in PSMA radioligand therapy (RLT) [25] might potentially benefit in the future from LLM support. In the present study, we used the LLM ChatGPT-4.0 [4]. Since its release in late 2022, this model has received much public and academic attention. It was initially trained by human feedback [26] and proximal policy optimisation algorithms [27]. Specifically in musculoskeletal (MSK) imaging, it has been tested previously for information extraction from image and text data [28,29,30]. The present study investigates the diagnostic accuracy of ChatGPT-4.0 in extracting correct diagnoses from multimodal MSK imaging text reports. The benchmark for comparison consists of the answers obtained from human experts. This study compares individual reader performance, as well as aggregated consensus performance. An earlier initial model for this topic has been presented at the 2025 convention of the Radiological Society of North America (RSNA) [31]. The present study also assesses clustering effects at the case and reader level (α_case, α_reader) and estimates effect sizes under consensus aggregation.

## 2. Method and Materials

The present study used a dataset of 23 open-source text cases of multimodal MSK imaging (see Supplementary S1). Human expert readers and the LLM answers were aggregated to consensus reading, and compared for performance evaluation.

### 2.1. Text Data

The 23 multimodal radiology text reports forming the dataset for the present study are attached in Supplementary S1 with online sources and medical sources of their true diagnosis whenever available. The dataset size of 23 cases was selected to maintain a manageable labelling workload for human readers. The texts were obtained from the MedPix collection (https://medpix.nlm.nih.gov), maintained by the National Institutes of Health (NIH). Cases were selected from the MedPix database using a structured sampling approach to ensure representation of core MSK diagnostic entities. Selection criteria included (i) availability of complete radiological report text, (ii) clearly established reference diagnoses from the source database, and (iii) inclusion of multiple imaging modalities (X-ray, ultrasound, CT, and magnetic resonance imaging (MRI)) to reflect multimodal practice. Additionally, a minimum number of cases per diagnostic category was ensured to avoid extreme class imbalance while maintaining feasibility for expert annotation. The resulting dataset represents a curated but diagnostically diverse sample of educational cases rather than a consecutive clinical cohort. Table 1 contains the diagnoses of the dataset, together with their frequency. All cases contained history and findings. Case 1, 2, and 3 in addition also contained an exam.

### 2.2. Surveying of ChatGPT, and of Human Readers

The text of each case from Supplementary S1 was given to ChatGPT-4.0 [4] (hereafter ‘LLM’) with a prompt requesting a primary (1st), and a secondary (2nd) diagnosis from a closed list of answering options; answering options being the 6 diagnoses of Table 1. Since LLM outputs are subject to statistical fluctuations, this step was repeated (*n* = 10) times. This approach implemented a six-option, two-choice question for the LLM. The exact prompt wording was:

For the radiology report above, please give from the 6 options below your most likely primary diagnosis, and alternative secondary diagnosis, please only give the respective diagnosis numbers:

[1] Osteosarcoma

[2] Abscess/Osteomyelitis

[3] Heterotopic ossification

[4] Myxoid Liposarcoma

[5] Lipoma

[6] Haemangioma

The prompt was deliberately designed as a constrained, closed-set classification task with 6 predefined diagnostic options and two ranked outputs (primary and secondary diagnosis). This structure was chosen to (i) ensure direct comparability between LLM outputs and human reader responses, (ii) reduce variability associated with free-text generation, and (iii) approximate clinical reasoning by allowing a differential diagnosis. Overall, this controlled prompt design prioritised internal validity and reproducibility over open-ended generative performance, aligning with the study’s objective of evaluating diagnostic extraction accuracy under standardised conditions.

The human participants (*n* = 10) were asked to give answers through Google Forms (Google LLC, Mountain View, CA, USA). Similar to the LLM, the human readers were presented with the six-option, two-choice question for each of the 23 MSK cases. The survey printout is attached in Supplementary S2. In the online version, the order of the questions was randomised for each human reader. Eight out of the 10 human readers were specialised in MSK radiology. The average work experience across all 10 human readers was 21.4 years. Nine out of the 10 human readers were board-certified radiologists with an average work experience of 17.6 years since that time point. The raw data with answers obtained from the LLM and the 10 human readers is contained in Supplementary S3.

**Figure 1:**
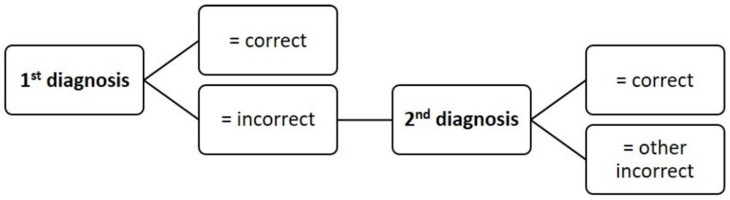
1st and 2nd diagnoses, with 2nd diagnosis replacing 1st when 1st is incorrect; intended as an option for the readers to provide differential diagnosis in alignment with real-world clinical workflows.

In the analyses of the present study, 1st answers were considered as first step. In a second step, incorrect 1st answers were replaced by 2nd answers giving “1st & 2nd”; see Figure 1. Through this, a broader range of answers with differential diagnosis was allowed.

### 2.3. Statistical Analyses

The answering data were analysed using Python (3.11.7) code, which is provided in Supplementary S4. The Python (3.11.7) code follows a structure used before in [30,32,33]. Answering data were analysed on individual reader level, as well as on aggregated reader consensus level. All significance testing was conducted at the alpha (α) level of 0.05.

**Figure 2:**
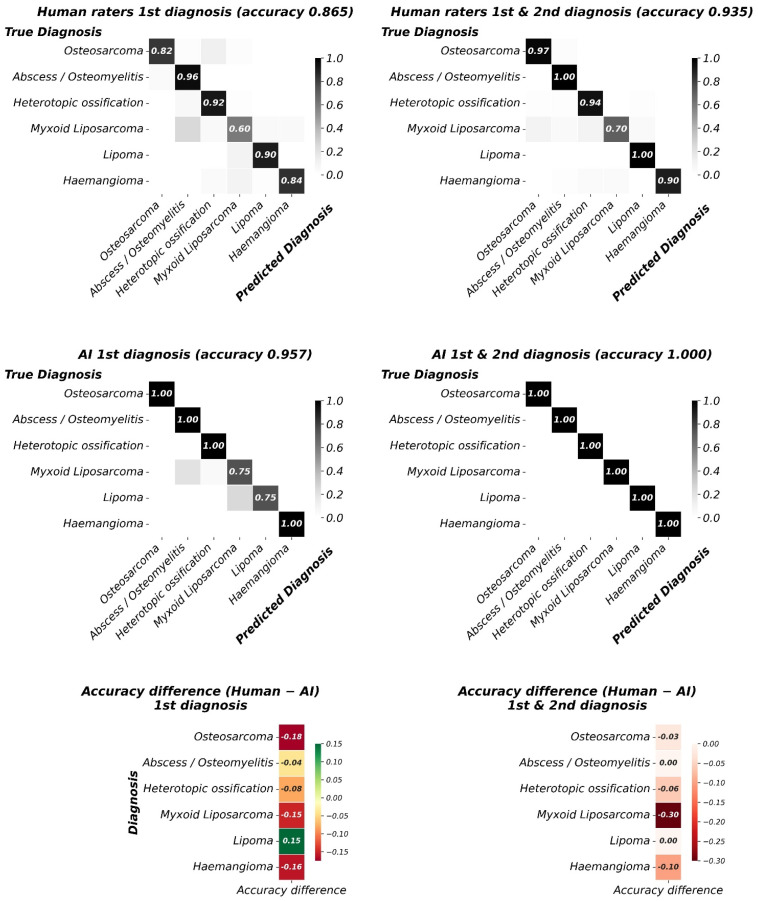
Individual reader level, not consensus-aggregated: confusion matrices for human readers (**top row**) and AI readers (**middle row**), as well as the obtained accuracy performance difference per diagnosis (**bottom row**).

Analyses on individual reader level. Raw accuracy proportions and descriptive 95% confidence intervals (95%–CI) were computed using Wilson’s method. Absolute differences in accuracy between human readers and AI were estimated using Newcombe’s method for independent proportions for descriptive comparison [34], as shown in Table 2. Normalised confusion matrices (cm) were computed per diagnosis [35] and are shown in Figure 2. Per-diagnosis, accuracy differences (Human–AI) were calculated directly from these counts and visualised in Figure 2, highlighting where humans outperformed the AI or vice versa.

Diagnostic performance was compared between human readers and AI using logistic generalised estimating equations (GEE) with a binomial distribution and logit link function and an exchangeable working correlation structure [36] (Table 2). The primary analysis clustered observations by case_id, assuming equal within-case correlation among ratings from the same case (α_case). A secondary descriptive GEE clustered by reader_id was used to estimate within-reader correlation (α_reader). The working correlation parameters α_case and α_reader were extracted to quantify case-related and reader-related variability. The primary inferential measure was the odds ratio (OR) for correct classification comparing AI with human readers, reported with robust 95%–CI and Wald *p*-values derived from the GEE model. To assess the impact of clustering on statistical precision, the design effect (DE) was calculated as:

(1) DE=1+(m−1)α

where m denotes the average number of ratings per case and α the case-related working correlation. An effective sample size was obtained by dividing the total number of observations by the design effect. In the presence of complete separation (i.e., 100% accuracy in the 1st & 2nd AI answering data), logistic models were not estimable; therefore, case-level majority consensus was derived, and paired comparisons were performed using exact McNemar testing [37] with Haldane correction for OR estimation.

**Figure 3:**
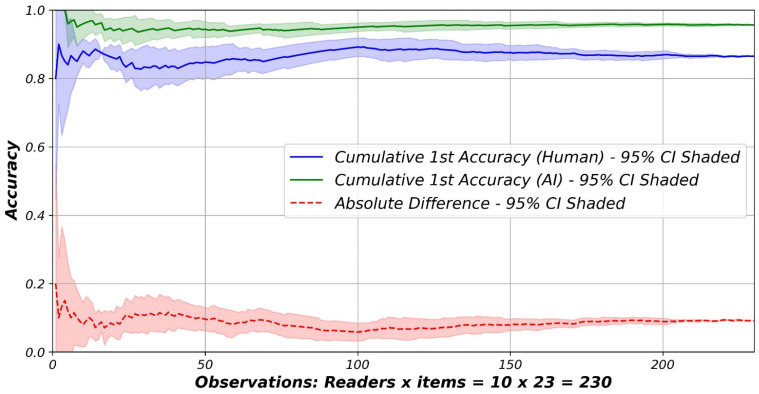
Individual reader level, not consensus-aggregated: model stability: cumulative 1st accuracy for human readers and AI converging towards constant, plotted over sample set, sample set shuffled 5 times, 95%–CI shaded.

Figure 3 plots for 5 iterations 1st accuracy of human answers and of AI answers over the growing dataset. The order of the data was shuffled 5 times.

Interrater reliability (IRR, Table 3) was assessed through the Python (3.11.7) package for interrater reliability Chance-corrected Agreement Coefficients (irrCAC) [38]. irrCAC allows the calculation of Gwet’s AC1 as well as Fleiss’ kappa. Gwet’s AC1 is specifically usable for imbalanced datasets; medical data being known for imbalance [39]. In addition, irrCAC can obtain the respective *p*-values. Those allow testing the Null Hypothesis (H0), which states that there is no agreement beyond what would be expected by chance alone. Both IRR variables take values in the interval [−1, 1] [40,41]. IRR was interpreted according to the benchmarks proposed by Landis and Koch [42]: values below 0.00 indicate poor agreement, 0.00–0.20 slight agreement, 0.21–0.40 fair agreement, 0.41–0.60 moderate agreement, 0.61–0.80 substantial agreement, and 0.81–1.00 almost perfect agreement.

**Consensus-aggregated paired comparison.** For consensus-based comparison, majority voting was performed at the case level separately for human readers and AI outputs. Consensus readings per case were obtained (Table 4) for each diagnosis in Table 1. Accuracy per diagnosis is given in Table 4, together with the accuracy difference (human–AI) per diagnosis. The 95%–CI for the accuracy difference between human and AI consensus readings is calculated using the McNemar-based standard error normal approximation; please see Supplementary S4.

The 2 × 2 contingency table was constructed summarising: (1) both correct, (2) both incorrect, (3) AI correct only, and (4) human correct only cases for performing exact McNemar testing at the case level (Table 5). The primary inferential parameter was the McNemar *p*-value based on exact binomial testing [37]. Overall agreement, proportions of both-correct and both-incorrect classifications, and the discordance rate were calculated using Wilson 95%–CI [34]. The discordance rate (proportion of cases in which human and AI consensus disagreed) was considered a descriptive measure of potential variability relevant to external validity.

### 2.4. Use of Generative AI

Generative AI tools (ChatGPT-5.2 [43], Claude Sonnet 4 [44], and Deepseek 3.1 [45]) were used for minor language optimisation, manuscript text editing, and Python (3.11.7) code debugging. All study design, data analysis, interpretation of results, and substantive content decisions were performed by the authors.

## 3. Results

A total of 460 observations were analysed for each comparison (23 cases × 20 ratings; 10 human readers and 10 AI revisions, Table 2). For 1st diagnoses, human accuracy was 0.865 (95%–CI 0.815–0.903) compared with 0.957 (95%–CI 0.922–0.976) for AI, yielding an absolute accuracy difference (human–AI) of −0.091 (95%–CI −0.145 to −0.040). Within-case correlation was 0.247, within-reader correlation −0.001 (Table 2). Cluster-adjusted logistic regression using case-level GEE demonstrated a significant advantage for AI (OR 3.427, 95%–CI 1.066–11.018; *p* = 3.87 × 10^−2^), resulting in a DE of 5.7 and an effective sample size of 80.7. When 1st and 2nd diagnoses were combined, human accuracy increased to 0.935 (95%–CI 0.895–0.960), while AI achieved 1.000 (95%–CI 0.984–1.000). The absolute difference was −0.065 (95%–CI −0.105 to −0.035). Within-case correlation decreased to 0.079, and within-reader correlation was 0.014. Due to complete separation, McNemar testing with Haldane correction was applied (OR 1.000, 95%–CI 0.561, 1.782; *p* = 1.00); the DE increased to 20.0, reducing the effective sample size to 23.0.

Figure 2 plots the cm for the AI and human readers, for 1st and 1st & 2nd diagnoses. The trends described above for the answering data accuracy also manifest visually. The answering data are mostly located on the main diagonal; greater convergence for the AI than for the human readers is seen. A perfect cm of perfect accuracy (1.000) is obtained for the AI when 1st & 2nd diagnoses are considered, with all data points lying exclusively on the main diagonal. The accuracy difference heatmaps (bottom row in Figure 2) show that AI outperformed human readers (red cells) across most diagnoses for the 1st diagnosis, with the exception of Lipoma (green cell) where human readers showed higher accuracy (human = 0.90, AI = 0.75, human–AI = 0.15). When considering 1st & 2nd diagnoses combined, AI performance was superior across all diagnoses (exclusively red cells), with the largest differences observed for Myxoid Liposarcoma (human = 0.70, AI = 1, human–AI = −0.30).

Gwet’s AC1 reaches marginally higher values than Fleiss’ kappa; see Table 3. This is what would be expected with the present data imbalance. By both measures, AI reaches a greater IRR for both 1st and 1st & 2nd answers. The 1st human IRR is substantial for Gwet’s AC1 (0.753) and Fleiss’ kappa (0.745). The remaining cases obtain almost perfect agreement. In all cases, the *p*-value of both measures is <<0.05; thus, the null hypothesis H0 that the agreement is a matter of pure chance can be rejected. The global IRR calculated between human and AI answers reaches a Gwet’s AC1 of 0.836 for 1st (almost perfect), and 0.927 for 1st & 2nd answers (almost perfect). The corresponding Fleiss’ kappa values are 0.830/0.925 (both almost perfect). For global IRR too, *p* << 0.05, and the null hypothesis H0 that the agreement is merely a matter of chance can be rejected.

Figure 3, which plots the 1st AI and 1st human accuracy over growing sample size together with the 95%–CI, demonstrates that 1st accuracy is largely independent from sample size, as soon as a size > 50 is reached. No substantial change in the 1st accuracy would be expected from increasing the study’s dataset.

At the case level (*n* = 23), the majority consensus agreement for the 1st diagnosis was 0.913 (95%–CI 0.732–0.976), with 21/23 cases correctly classified by both humans and AI (Table 5). Discordance occurred in 2/23 cases (0.087, 95%–CI 0.024–0.268), consisting of one AI-only and one human-only correct classification; McNemar’s exact test showed no significant difference (*p* = 1.00). Per-diagnosis results (Table 4) demonstrated perfect consensus accuracy (1.000) for 4/6 entities for both methods. Differences were observed for Myxoid Liposarcoma (human 0.500 vs. AI 1.000) and Lipoma (human 1.000 vs. AI 0.500); however, confidence intervals were wide and crossed zero. When 1st and 2nd diagnoses were combined, both human and AI consensus achieved 23/23 correct classifications (agreement 1.000, 95%–CI 0.857–1.000), with no discordant cases and no significant difference (McNemar *p* = 1.00) (Table 5).

## 4. Discussion

The initial motivation for the present study was to investigate the potential of large language models (LLMs) to assist healthcare staff in administrative and documentation tasks. Increasing demand for radiological services and a projected workforce shortage make efficiency gains highly relevant [2]. Previous work has evaluated LLMs in MSK imaging contexts [28,29,30]. The present study extends this literature by focusing specifically on diagnostic extraction from structured text reports and by evaluating both individual-reader and consensus-aggregated performance.

At the individual reader level, the LLM demonstrated higher 1st diagnosis accuracy than human readers (0.957 vs. 0.865), with a significant cluster-adjusted OR of 3.43. When 1st and 2nd diagnoses were combined, human performance improved (0.935), while the LLM achieved perfect accuracy.

Based on the consensus McNemar contingency table (Table 5), the between-method effect at the case level appears small. Only 2 of 23 cases (0.087) were discordant for the 1st diagnosis (one AI-only correct and one human-only correct), yielding McNemar *p* = 1.00 and overall agreement of 0.913 (95%–CI 0.732–0.976). This symmetric discordance suggests that large performance differences between AI and human consensus are unlikely in similar structured diagnostic settings. Even under plausible variation in case mix, the observed data support the expectation that future external validations would likely reveal single-digit percentage-point differences at the consensus level rather than large effect sizes. While modest individual-reader differences (≈6–9% absolute accuracy in Table 2) may persist, the case-level aggregation results indicate that substantial or clinically dramatic superiority of one method over the other is improbable based on the current evidence.

This mirrors findings from AI-supported chest radiograph interpretation, where hybrid human–AI workflows improved or stabilised diagnostic performance without displacing clinician oversight [8,13,31,46,47]. The IRR analysis supports this interpretation. AI responses demonstrated almost perfect agreement (Gwet’s AC1 ≥ 0.938), and global agreement between human and AI classifications reached 0.836 (1st diagnosis) and 0.927 (1st & 2nd), indicating substantial-to-almost-perfect concordance. Such convergence suggests that LLMs may function effectively as consistency-enhancing tools within structured diagnostic tasks.

An important methodological insight arises from the clustering analysis. The within-case correlation (α case = 0.247 for 1st diagnoses, Table 2) was substantially larger than the within-reader correlation (α reader ≈ 0). This indicates that variability in diagnostic correctness was driven primarily by case characteristics rather than reader-dependent differences. Consequently, statistical precision is improved more efficiently by increasing the number of cases than by increasing the number of readers, once a reasonable expert panel is included. This is reflected in the design effect (DE = 5.7), which reduced the effective sample size to 80.7 despite 460 observations. For future validation studies, expanding the spectrum and number of cases is therefore more critical than recruiting additional readers.

The human reader cohort consisted predominantly of MSK specialists (8/10), complemented by two non-specialised radiologists, with high overall experience (mean 21.4 years; 9/10 board-certified). Given the limited sample size, formal subgroup comparisons were not statistically powered; however, descriptive analysis together with clustering results indicated minimal reader-dependent variability (α_reader ≈ 0, Table 2), suggesting that differences between radiologist subgroups did not materially influence overall performance. Instead, diagnostic variability was primarily driven by case characteristics, as reflected by the substantially higher within-case correlation (α_case = 0.247).

Regarding generalizability, the cumulative accuracy analysis suggests that accuracy stabilises once sample sizes exceed approximately 50 observations. This argues against the observed effect being purely a small-sample fluctuation. Nevertheless, the present dataset consists of curated educational cases (https://medpix.nlm.nih.gov). In more heterogeneous real-world datasets—including ambiguous reports, incomplete documentation, or atypical imaging findings—absolute diagnostic accuracy would likely decrease for both human readers and AI. Such regression toward lower overall performance would be expected in external datasets of greater complexity. Importantly, the present data provide no indication that the relative performance relationship would necessarily reverse; rather, both systems are likely to experience similar performance attenuation. Prospective validation on consecutive clinical cases remains essential.

This study has several important limitations. The relatively small dataset (23 cases) limits external validity, as it may not fully capture the heterogeneity, ambiguity, and spectrum of findings encountered in routine clinical practice, and may therefore lead to optimistic estimates of diagnostic performance. The use of curated MedPix educational cases introduces potential selection bias and may overrepresent well-defined (“textbook”) presentations, limiting generalizability to real-world clinical settings. Reproducibility remains uncertain given that LLMs are proprietary black-box systems subject to version changes or discontinuation without notice, making long-term replication studies challenging; furthermore, the lack of transparency regarding training data, model updates, and inference parameters may result in non-deterministic outputs even under identical input conditions. Finally, the controlled nature of this evaluation, using structured text reports with predefined diagnostic options, does not capture the full spectrum of challenges in clinical radiology, including integration with imaging data, patient history synthesis, and communication of uncertainty.

## 5. Conclusions

Large language models can achieve diagnostic accuracy comparable to that of experienced radiologists in the interpretation of structured MSK text reports. At the individual level, the LLM demonstrated superior 1st diagnosis accuracy, while consensus aggregation eliminated statistically significant differences, suggesting that in clinical practice, AI may provide value primarily as a decision-support tool that supports individual readers and reduces variability, rather than replacing established multi-reader or consensus-based diagnostic workflows. Variability analysis indicates that future studies will benefit more from increasing case numbers than reader numbers, as diagnostic variability is predominantly case-driven.

In the future, diagnostically heterogeneous real-world datasets will likely reduce absolute performance for both humans and AI. However, the current data suggest that relative performance differences are unlikely to reverse, supporting the plausibility of LLMs as assistive tools in supervised human–AI workflows. Their role should be viewed as augmentative rather than autonomous.

While barriers remain—including data security concerns, limitations in areas such as MRI reconstruction [48,49] and supplementary of ref. [48], regulatory considerations [50], and the potential risk of professional deskilling [51]—the present findings contribute to a growing body of evidence that hybrid human–AI systems may help address capacity challenges in radiology [8,13,46,47]. Robust prospective validation across larger and diagnostically diverse datasets will be required to determine the true generalizability and long-term clinical impact of such systems.

## Figures and Tables

**Table 1:** Diagnoses of the 23 total study cases with frequency.

Diagnosis	Frequency
Osteosarcoma	4
Abscess/Osteomyelitis	5
Heterotopic ossification	5
Myxoid Liposarcoma	2
Lipoma	2
Haemangioma	5
Total	23

**Table 2:** Individual-reader performance; GEE for 1st diagnosis and case-level exact McNemar (Haldane correction) for 1st & 2nd diagnosis due to complete separation.

Diagnosis Type	1st	1st & 2nd
Total observations	23 × (10 + 10) = 460	23 × (10 + 10) = 460
Samples	23	23
Human readers + AI revisions	10 + 10 = 20	10 + 10 = 20
Reader performance:		
Human accuracy [95%–CI]	0.865 [0.815, 0.903]	0.935 [0.895, 0.960]
AI accuracy [95%–CI]	0.957 [0.922, 0.976]	1.000 [0.984, 1.000]
Absolute accuracy difference: human–AI [95%–CI]	−0.091 [−0.145, −0.040]	−0.065 [−0.105, −0.035]
Clustering parameters:		
α_reader	−0.001	0.014
α_case	0.247	0.079
Inference method:		
Separation	none	complete
Applied statistical method	GEE (case-clustered)	Exact McNemar (Haldane correction)
Odds ratio [95%–CI]	3.427 [1.066, 11.018]	1.000 [0.561, 1.782]
*p*-value	3.87 × 10^−2^	1.00
Design effect (DE)	5.7	20.0
Effective sample size	80.7	23.0

Statistical note: Working correlation parameters (α_case, α_reader) were estimated from GEE models for descriptive assessment of clustering structure (1st), while inferential statistics were derived from McNemar testing in the presence of separation (1st & 2nd).

**Table 3:** Individual reader IRR in the obtained answering data (not consensus reading, all *p*-values < alpha = 0.05, highlighted).

IRR	Human 1st	Human 1st & 2nd	AI 1st	AI 1st & 2nd
Fleiss’ kappa	0.745	0.860	0.935	1.000
interpretation	Substantial	almost perfect	almost perfect	almost perfect
Fleiss’ kappa *p*-value	1.36 × 10^−11^	5.77 × 10^−14^	4.44 × 10^−16^	0.00
Gwet’s AC1	0.753	0.865	0.938	1.000
interpretation	substantial	almost perfect	almost perfect	almost perfect
Gwet’s AC1 *p*-value	1.02 × 10^−11^	3.71 × 10^−14^	2.22 × 10^−16^	0.00

**Table 4:** Consensus-aggregated results: majority consensus for human readers and AI, after both 1^st^ diagnosis and 1^st^ and 2^nd^ diagnosis.

	Cases—Majority Consensus			Accuracy				
1^st^ diagnosis	Count	Human correct	AI correct	Human	AI	Human—AI	95%-CI lower bound	95%—CI upper bound
Osteosarcoma	4	4	4	1.000	1.000	0.000	Perfect agreement	Perfect agreement
Abscess/Osteomyelitis	5	5	5	1.000	1.000	0.000	Perfect agreement	Perfect agreement
Heterotopic ossification	5	5	5	1.000	1.000	0.000	Perfect agreement	Perfect agreement
Myxoid Liposarcoma	2	1	2	0.500	1.000	−0.500	−1.480	0.480
Lipoma	2	2	1	1.000	0.500	0.500	−0.480	1.480
Haemangioma	5	5	5	1.000	1.000	0.000	Perfect agreement	Perfect agreement
	Cases—majority consensus			Accuracy				
1^st^ and 2^nd^ diagnosis	Count	Human correct	AI correct	Human	AI	Human—AI	95%-CI lower bound	95%-CI upper bound
Osteosarcoma	4	4	4	1.000	1.000	0.000	Perfect agreement	Perfect agreement
Abscess/Osteomyelitis	5	5	5	1.000	1.000	0.000	Perfect agreement	Perfect agreement
Heterotopic ossification	5	5	5	1.000	1.000	0.000	Perfect agreement	Perfect agreement
Myxoid Liposarcoma	2	2	2	1.000	1.000	0.000	Perfect agreement	Perfect agreement
Lipoma	2	2	2	1.000	1.000	0.000	Perfect agreement	Perfect agreement
Haemangioma	5	5	5	1.000	1.000	0.000	Perfect agreement	Perfect agreement

**Table 5:** Consensus-aggregated paired comparison: contingency tables and McNemar significance testing for majority consensus.

Diagnosis Type	1st Diagnosis			1st and 2nd Diagnosis		
Samples	23			23		
Contingency Table		AI Wrong	AI Correct		AI Wrong	AI Correct
	Human Wrong	0	1	Human Wrong	0	0
	Human Correct	1	21	Human Correct	0	23
Agreement [95%–CI]	0.913 [0.732, 0.976]			1.000 [0.857, 1.000]		
Both Correct [95%–CI]	0.913 [0.732, 0.976]			1.000 [0.857, 1.000]		
Both incorrect [95%–CI]	0.000 [0.000, 0.143]			0.000 [0.000, 0.143]		
Discordance Rate [95%–CI]	0.087 [0.024, 0.268]			0.000 [0.000, 0.143]		
McNemar *p*-value	1.00			1.00		

## Data Availability

The original contributions presented in this study are included in the Supplementary Material. Further inquiries can be directed to the corresponding author.

## Supplementary Materials

The raw data used for the present study is deposited permanently under https://doi.org/10.6084/m9.figshare.28664792, S1: Radiology reports with original source and true diagnosis (.pdf); S2: Survey for human readers implemented in Google-Forms (.pdf); S3: Study answers collected from human readers and from ChatGPT-4.0 (.xlsx); S4: Python code for data evaluation (.py).
